# Bis(μ-4-fluoro-2,6-diformyl­phenolato)bis­[diaqua­nickel(II)] dichloride

**DOI:** 10.1107/S1600536810016284

**Published:** 2010-05-19

**Authors:** Yin Zheng, Shi-Rong Li, Hong Zhou, Zhi-Quan Pan, Yi-Zhi Li

**Affiliations:** a Key Laboratory for Green Chemical Processes of the Ministry of Education, Wuhan Institute of Technology, Wuhan 430073, People’s Republic of China; b Hubei Key Laboratory of Biologic Resources Protection and Utilization, Hubei Institute for Nationalities, Wuhan Institute of Technology, Enshi 445000, People’s Republic of China; c State Key Laboratory of Coordination Chemistry, Coordination Chemistry Institute, Nanjing University, Nanjing 210093, People’s Republic of China

## Abstract

In the title dinuclear nickel(II) complex, [Ni_2_(C_8_H_4_FO_3_)_2_(H_2_O)_4_]Cl_2_, synthesized by the reaction between 4-fluoro-2,6-diformyl­phenol and nickel(II) chloride in methanol, the coordination cation is located on an inversion center and the Ni^II^ atom adopts a slightly distorted octa­hedral coordination geometry. The two Ni atoms are bridged by two phenolate O atoms and the intra­molecular Ni⋯Ni distance is 3.0751 (9) Å. The crystal structure is stabilized by O—H⋯Cl hydrogen bonds.

## Related literature

For the synthesis of related compounds and their properties, see: Thompson *et al.* (1996 ▶); Zhou *et al.* (2005 ▶); Raimondi *et al.* (2004 ▶); Taniguchi (1984 ▶); Mohanta *et al.* (1998 ▶); Wang *et al.* (1997 ▶). For related structures, see: Adhikary *et al.* (1987 ▶); Zhou *et al.* (2007 ▶).
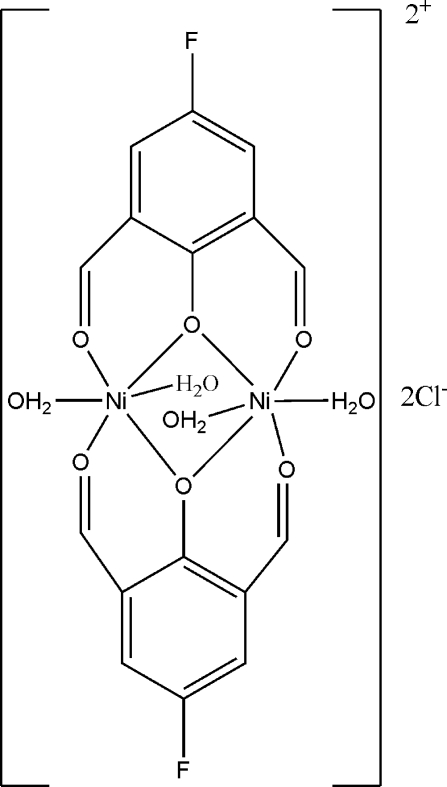


## Experimental

### 

#### Crystal data

[Ni_2_(C_8_H_4_FO_3_)_2_(H_2_O)_4_]Cl_2_
*M* *_r_* = 594.61
Monoclinic, 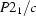

*a* = 8.3299 (14) Å
*b* = 13.576 (2) Å
*c* = 9.9965 (17) Å
β = 114.623 (3)°
*V* = 1027.6 (3) Å^3^
*Z* = 2
Mo *K*α radiation
μ = 2.16 mm^−1^
*T* = 291 K
0.26 × 0.22 × 0.20 mm

#### Data collection

Bruker SMART APEX CCD diffractometer
Absorption correction: multi-scan (*SADABS*; Sheldrick, 1996 ▶) *T*_min_ = 0.603, *T*_max_ = 0.672
5827 measured reflections
2018 independent reflections
1708 reflections with *I* > 2σ(*I*)
*R*_int_ = 0.041

#### Refinement

*R*[*F* ^2^ > 2σ(*F* ^2^)] = 0.051
*wR*(*F* ^2^) = 0.119
*S* = 1.05
2018 reflections
157 parameters
4 restraints
H atoms treated by a mixture of independent and constrained refinement
Δρ_max_ = 0.42 e Å^−3^
Δρ_min_ = −0.94 e Å^−3^

### 

Data collection: *SMART* (Bruker, 2007 ▶); cell refinement: *SAINT* (Bruker, 2007 ▶); data reduction: *SAINT*; program(s) used to solve structure: *SHELXTL* (Sheldrick, 2008 ▶); program(s) used to refine structure: *SHELXTL*; molecular graphics: *SHELXTL*; software used to prepare material for publication: *SHELXTL*.

## Supplementary Material

Crystal structure: contains datablocks global, I. DOI: 10.1107/S1600536810016284/gk2267sup1.cif

Structure factors: contains datablocks I. DOI: 10.1107/S1600536810016284/gk2267Isup2.hkl

Additional supplementary materials:  crystallographic information; 3D view; checkCIF report

## Figures and Tables

**Table 1:** Hydrogen-bond geometry (Å, °)

*D*—H⋯*A*	*D*—H	H⋯*A*	*D*⋯*A*	*D*—H⋯*A*
O4—H4*A*⋯Cl1	0.85 (5)	2.44 (3)	3.198 (4)	149 (6)
O4—H4*B*⋯Cl1^i^	0.85 (5)	2.45 (3)	3.241 (4)	154 (5)
O5—H5*C*⋯Cl1^ii^	0.85 (2)	2.61 (4)	3.313 (4)	141 (5)
O5—H5*A*⋯Cl1^iii^	0.86 (6)	2.39 (4)	3.101 (4)	142 (5)

Symmetry codes: (i) 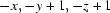; (ii) 
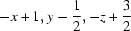
; (iii) 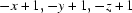.

**Fig. 1.**
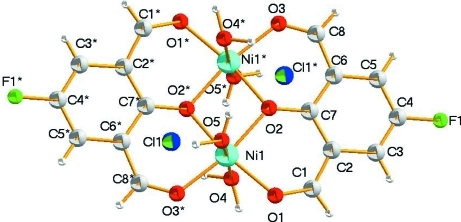
A view of the title complex, showing the labeling of the non-H atoms and 30% probability displacement ellipsoids. Atoms with the suffix (*) are generated by the symmetry operation 1-x, 1-y, 1-z.

## supplementary crystallographic information

### Comment

Phenoxide-bridged dinuclear complexes have been extensively studied for several decades, most of them were derived from the cyclocondensation of 2,6-diformyl-4-R-phenol and alkyldiamine in the presence of metal ions (Thompson *et al.*,1996; Zhou *et al.*, 2005; Raimondi *et al.*, 2004).With short distances between the two metal ions in the complexes, they show special electrical and magnetic properties (Mohanta *et al.*, 1998; Wang *et al.*, 1997). Adhikary *et al.* reported a phenoxide-bridged dinuclear nickel(II) complex, obtained directly from the mixture of 2,6-diformyl-4-methyl-phenol and nickel(II) perchlorate (Adhikary *et al.*, 1987). Here we report the crystal structure of a new dinuclear Ni^II^ complex with fluorine substituent in the phenyl ring. The diference between the title complex and the one Adhikary reported is that they have different substituents in the phenyl ring and different counter-anions.

The coordination cation consists of two 2,6-diformyl-4-flurophenolate ligands, four water molecules, two Ni^II^ ions (Fig. 1). The chlorine ions do not participate in coordination to the Ni atoms. Each Ni atom has a slightly distorted octahedral coordination geometry and it deviates from the equatorial plane defined by four coordinating oxygen atoms of the organic ligand by 0.0266 (4) Å. The axial positions are occupied by two water molecules with Ni–O distances of 2.057 (4) Å and 2.067 (4) Å.The Ni—O distance in the basal plane is in the range of 1.995 (4) Å - 2.019 (3) Å. The presence of the two bridging phenolate O atoms gives rise to a short metal-metal contact of 3.0751 (9) Å that is slightly longer than those of binuclear nickel(II) complexes with macrocyclic phenoxo-bridging ligands (Zhou *et al.*, 2007).

### Experimental

2, 6-Diformyl-4-fluorophenol was prepared according to the literature method (Taniguchi, 1984). To a solution of 2,6-diformyl-4-fluorinphenol (1 mmol, 0.17 g) in absolute methanol (10 ml) was added a methanol solution (10 ml) containing NiCl_2_2H_2_O (1 mmol, 0.17 g). The solution was stirred vigorously for 24 h at room temperature and filtrated. The dark-green block-shaped crystals suitable for X-ray diffraction analysis were obtained by slow evaporation of solvent over a period of two weeks.

### Refinement

The H atoms of water molecules were found in a difference Fourier map, and the O—H distances were restrained to 0.85 (1) Å; their temperature factor was set to 1.2*U*_eq_(O). All other H atoms were placed in calculated positions with C—H = 0.93 Å and included in the refinement in the riding-model approximation with *U*(H) set to 1.2*U*_eq_(C).

### Crystal data

**Table:**

[Ni_2_(C_8_H_4_FO_3_)_2_(H_2_O)_4_]Cl_2_	*F*(000) = 600
*M**_r_* = 594.61	*D*_x_ = 1.922 Mg m^−^^3^
Monoclinic, *P*2_1_/*c*	Mo *K*α radiation, λ = 0.71073 Å
Hall symbol: -P 2ybc	Cell parameters from 3902 reflections
*a* = 8.3299 (14) Å	θ = 2.2–28.0°
*b* = 13.576 (2) Å	µ = 2.16 mm^−^^1^
*c* = 9.9965 (17) Å	*T* = 291 K
β = 114.623 (3)°	Block, green
*V* = 1027.6 (3) Å^3^	0.26 × 0.22 × 0.20 mm
*Z* = 2	

### Data collection

**Table:**

Bruker SMART APEX CCD diffractometer	2018 independent reflections
Radiation source: sealed tube	1708 reflections with *I* > 2σ(*I*)
graphite	*R*_int_ = 0.041
phi and ω scans	θ_max_ = 26.0°, θ_min_ = 2.7°
Absorption correction: multi-scan (*SADABS*; Sheldrick, 1996)	*h* = −10→7
*T*_min_ = 0.603, *T*_max_ = 0.672	*k* = −15→16
5827 measured reflections	*l* = −8→12

### Refinement

**Table:**

Refinement on *F*^2^	Primary atom site location: structure-invariant direct methods
Least-squares matrix: full	Secondary atom site location: difference Fourier map
*R*[*F*^2^ > 2σ(*F*^2^)] = 0.051	Hydrogen site location: inferred from neighbouring sites
*wR*(*F*^2^) = 0.119	H atoms treated by a mixture of independent and constrained refinement
*S* = 1.05	*w* = 1/[σ^2^(*F*_o_^2^) + (0.06*P*)^2^ + 1.99*P*] where *P* = (*F*_o_^2^ + 2*F*_c_^2^)/3
2018 reflections	(Δ/σ)_max_ < 0.001
157 parameters	Δρ_max_ = 0.42 e Å^−^^3^
4 restraints	Δρ_min_ = −0.94 e Å^−^^3^

### Special details

**Table:**

Geometry. All esds (except the esd in the dihedral angle between two l.s. planes) are estimated using the full covariance matrix. The cell esds are taken into account individually in the estimation of esds in distances, angles and torsion angles; correlations between esds in cell parameters are only used when they are defined by crystal symmetry. An approximate (isotropic) treatment of cell esds is used for estimating esds involving l.s. planes.
Refinement. Refinement of *F*^2^ against ALL reflections. The weighted *R*-factor wR and goodness of fit *S* are based on *F*^2^, conventional *R*-factors *R* are based on *F*, with *F* set to zero for negative *F*^2^. The threshold expression of *F*^2^ > σ(*F*^2^) is used only for calculating *R*-factors(gt) etc. and is not relevant to the choice of reflections for refinement. *R*-factors based on *F*^2^ are statistically about twice as large as those based on *F*, and *R*- factors based on ALL data will be even larger.

### Fractional atomic coordinates and isotropic or equivalent isotropic displacement parameters (Å^2^)

**Table:**

	*x*	*y*	*z*	*U*_iso_*/*U*_eq_	
C1	0.2077 (6)	0.3085 (3)	0.2584 (5)	0.0349 (11)	
H1	0.1159	0.2647	0.2095	0.042*	
C2	0.2373 (6)	0.3811 (3)	0.1689 (5)	0.0281 (9)	
C3	0.1336 (6)	0.3705 (3)	0.0166 (5)	0.0331 (10)	
H3	0.0559	0.3178	−0.0182	0.040*	
C4	0.1475 (7)	0.4376 (4)	−0.0792 (5)	0.0401 (12)	
C5	0.2548 (7)	0.5179 (4)	−0.0329 (5)	0.0370 (11)	
H5	0.2609	0.5630	−0.1007	0.044*	
C6	0.3552 (6)	0.5318 (3)	0.1172 (5)	0.0276 (9)	
C7	0.3474 (5)	0.4643 (3)	0.2223 (5)	0.0228 (8)	
C8	0.4651 (6)	0.6193 (4)	0.1539 (6)	0.0356 (11)	
H8	0.4680	0.6545	0.0752	0.043*	
Cl1	0.16724 (17)	0.66791 (9)	0.64475 (15)	0.0406 (3)	
F1	0.0499 (5)	0.4245 (3)	−0.2256 (3)	0.0555 (9)	
Ni1	0.43045 (7)	0.40202 (4)	0.53212 (6)	0.02194 (18)	
O1	0.2874 (4)	0.2955 (2)	0.3923 (4)	0.0318 (7)	
O2	0.4438 (4)	0.4760 (2)	0.3627 (3)	0.0249 (6)	
O3	0.5544 (5)	0.6519 (2)	0.2770 (4)	0.0351 (8)	
O4	0.1900 (5)	0.4629 (3)	0.4960 (4)	0.0415 (8)	
H4A	0.201 (8)	0.502 (4)	0.565 (5)	0.050*	
H4B	0.116 (6)	0.417 (3)	0.484 (7)	0.050*	
O5	0.6594 (5)	0.3234 (3)	0.5759 (4)	0.0402 (8)	
H5C	0.725 (7)	0.313 (4)	0.6662 (18)	0.048*	
H5A	0.717 (7)	0.354 (4)	0.535 (6)	0.048*	

### Atomic displacement parameters (Å^2^)

**Table:**

	*U*^11^	*U*^22^	*U*^33^	*U*^12^	*U*^13^	*U*^23^
C1	0.034 (2)	0.032 (2)	0.034 (3)	−0.0109 (19)	0.008 (2)	−0.002 (2)
C2	0.024 (2)	0.025 (2)	0.035 (2)	−0.0003 (17)	0.0122 (18)	−0.0051 (18)
C3	0.028 (2)	0.032 (2)	0.034 (3)	0.0002 (19)	0.0079 (19)	−0.010 (2)
C4	0.050 (3)	0.042 (3)	0.019 (2)	0.012 (2)	0.006 (2)	−0.001 (2)
C5	0.055 (3)	0.035 (3)	0.018 (2)	0.001 (2)	0.012 (2)	0.0028 (19)
C6	0.031 (2)	0.027 (2)	0.024 (2)	−0.0020 (17)	0.0109 (17)	0.0029 (17)
C7	0.0206 (19)	0.0228 (19)	0.028 (2)	0.0044 (15)	0.0127 (17)	0.0059 (17)
C8	0.039 (3)	0.036 (3)	0.036 (3)	0.004 (2)	0.020 (2)	0.011 (2)
Cl1	0.0417 (7)	0.0394 (6)	0.0449 (7)	0.0017 (5)	0.0222 (6)	−0.0033 (5)
F1	0.066 (2)	0.0570 (19)	0.0244 (15)	−0.0102 (17)	−0.0007 (14)	−0.0047 (14)
Ni1	0.0241 (3)	0.0209 (3)	0.0230 (3)	−0.0028 (2)	0.0120 (2)	0.0000 (2)
O1	0.0358 (16)	0.0266 (16)	0.0336 (18)	−0.0065 (13)	0.0149 (14)	−0.0018 (13)
O2	0.0303 (15)	0.0256 (14)	0.0188 (14)	−0.0044 (12)	0.0103 (12)	0.0019 (11)
O3	0.0455 (19)	0.0349 (18)	0.0282 (18)	−0.0055 (15)	0.0186 (15)	0.0046 (14)
O4	0.0335 (18)	0.040 (2)	0.053 (2)	0.0004 (15)	0.0196 (17)	−0.0023 (17)
O5	0.0359 (18)	0.051 (2)	0.0361 (19)	0.0158 (16)	0.0178 (15)	0.0147 (17)

### Geometric parameters (Å, °)

**Table:**

C1—O1	1.234 (6)	C8—O3	1.226 (6)
C1—C2	1.421 (7)	C8—H8	0.9300
C1—H1	0.9300	Ni1—O3^i^	1.998 (3)
C2—C3	1.410 (6)	Ni1—O2^i^	2.007 (3)
C2—C7	1.412 (6)	Ni1—O2	2.012 (3)
C3—C4	1.361 (7)	Ni1—O1	2.019 (3)
C3—H3	0.9300	Ni1—O4	2.054 (4)
C4—F1	1.358 (5)	Ni1—O5	2.067 (3)
C4—C5	1.364 (7)	O2—Ni1^i^	2.007 (3)
C5—C6	1.393 (6)	O3—Ni1^i^	1.998 (3)
C5—H5	0.9300	O4—H4A	0.85 (5)
C6—C7	1.416 (6)	O4—H4B	0.85 (5)
C6—C8	1.450 (6)	O5—H5C	0.85 (2)
C7—O2	1.304 (5)	O5—H5A	0.86 (6)
			
O1—C1—C2	128.7 (4)	O3^i^—Ni1—O2	169.53 (13)
O1—C1—H1	115.6	O2^i^—Ni1—O2	80.18 (13)
C2—C1—H1	115.6	O3^i^—Ni1—O1	100.54 (14)
C3—C2—C1	114.8 (4)	O2^i^—Ni1—O1	169.22 (12)
C3—C2—C7	119.9 (4)	O2—Ni1—O1	89.91 (13)
C1—C2—C7	125.0 (4)	O3^i^—Ni1—O4	89.11 (15)
C4—C3—C2	119.9 (4)	O2^i^—Ni1—O4	91.02 (14)
C4—C3—H3	120.1	O2—Ni1—O4	92.46 (14)
C2—C3—H3	120.1	O1—Ni1—O4	85.14 (14)
F1—C4—C3	118.9 (5)	O3^i^—Ni1—O5	85.59 (15)
F1—C4—C5	118.9 (5)	O2^i^—Ni1—O5	94.66 (15)
C3—C4—C5	122.2 (4)	O2—Ni1—O5	93.79 (13)
C4—C5—C6	119.2 (5)	O1—Ni1—O5	90.19 (15)
C4—C5—H5	120.4	O4—Ni1—O5	172.19 (15)
C6—C5—H5	120.4	C1—O1—Ni1	123.0 (3)
C5—C6—C7	121.3 (4)	C7—O2—Ni1^i^	128.1 (3)
C5—C6—C8	114.5 (4)	C7—O2—Ni1	128.3 (3)
C7—C6—C8	124.1 (4)	Ni1^i^—O2—Ni1	99.82 (13)
O2—C7—C2	121.1 (4)	C8—O3—Ni1^i^	126.6 (3)
O2—C7—C6	121.5 (4)	Ni1—O4—H4A	110 (4)
C2—C7—C6	117.4 (4)	Ni1—O4—H4B	109 (4)
O3—C8—C6	127.3 (4)	H4A—O4—H4B	114 (6)
O3—C8—H8	116.4	Ni1—O5—H5C	116 (4)
C6—C8—H8	116.4	Ni1—O5—H5A	108 (4)
O3^i^—Ni1—O2^i^	89.45 (13)	H5C—O5—H5A	111 (6)
			
O1—C1—C2—C3	174.3 (5)	O3^i^—Ni1—O1—C1	161.0 (4)
O1—C1—C2—C7	−11.6 (8)	O2^i^—Ni1—O1—C1	3.4 (9)
C1—C2—C3—C4	178.2 (5)	O2—Ni1—O1—C1	−19.6 (4)
C7—C2—C3—C4	3.7 (7)	O4—Ni1—O1—C1	72.9 (4)
C2—C3—C4—F1	178.3 (4)	O5—Ni1—O1—C1	−113.4 (4)
C2—C3—C4—C5	−2.4 (8)	C2—C7—O2—Ni1^i^	−166.2 (3)
F1—C4—C5—C6	180.0 (5)	C6—C7—O2—Ni1^i^	16.8 (6)
C3—C4—C5—C6	0.7 (8)	C2—C7—O2—Ni1	−12.6 (5)
C4—C5—C6—C7	−0.3 (8)	C6—C7—O2—Ni1	170.5 (3)
C4—C5—C6—C8	179.7 (5)	O3^i^—Ni1—O2—C7	−167.1 (7)
C3—C2—C7—O2	179.7 (4)	O2^i^—Ni1—O2—C7	−159.3 (4)
C1—C2—C7—O2	5.8 (7)	O1—Ni1—O2—C7	16.5 (3)
C3—C2—C7—C6	−3.3 (6)	O4—Ni1—O2—C7	−68.7 (3)
C1—C2—C7—C6	−177.1 (4)	O5—Ni1—O2—C7	106.7 (3)
C5—C6—C7—O2	178.6 (4)	O3^i^—Ni1—O2—Ni1^i^	−7.8 (8)
C8—C6—C7—O2	−1.4 (7)	O2^i^—Ni1—O2—Ni1^i^	0.0
C5—C6—C7—C2	1.6 (6)	O1—Ni1—O2—Ni1^i^	175.74 (14)
C8—C6—C7—C2	−178.4 (4)	O4—Ni1—O2—Ni1^i^	90.61 (15)
C5—C6—C8—O3	173.9 (5)	O5—Ni1—O2—Ni1^i^	−94.08 (16)
C7—C6—C8—O3	−6.1 (8)	C6—C8—O3—Ni1^i^	−2.9 (7)
C2—C1—O1—Ni1	21.3 (7)		

Symmetry codes: (i) −*x*+1, −*y*+1, −*z*+1.

### Hydrogen-bond geometry (Å, °)

**Table:**

*D*—H···*A*	*D*—H	H···*A*	*D*···*A*	*D*—H···*A*
O4—H4A···Cl1	0.85 (5)	2.44 (3)	3.198 (4)	149 (6)
O4—H4B···Cl1^ii^	0.85 (5)	2.45 (3)	3.241 (4)	154 (5)
O5—H5C···Cl1^iii^	0.85 (2)	2.61 (4)	3.313 (4)	141 (5)
O5—H5A···Cl1^i^	0.86 (6)	2.39 (4)	3.101 (4)	142 (5)

Symmetry codes: (ii) −*x*, −*y*+1, −*z*+1; (iii) −*x*+1, *y*−1/2, −*z*+3/2; (i) −*x*+1, −*y*+1, −*z*+1.
